# Long-term outcome and relapse patterns in Ewing sarcoma patients with extensive lung/pleural metastases after a complete response to systemic therapy

**DOI:** 10.1186/s12885-022-09618-w

**Published:** 2022-05-06

**Authors:** Jie Xu, Xin Zhi, Lu Xie, Xin Sun, Xingyu Liu, Kuisheng Liu, Wei Guo

**Affiliations:** 1 Musculoskeletal Tumor Center, Peking University People’s Hospital, 11 Xizhimen South Street, Beijing, China; 2 Radiology Department, Peking University People’s Hospital, 11 Xizhimen South Street, Beijing, China

**Keywords:** Ewing sarcoma, Metastases, Relapse, Complete remission

## Abstract

**Background:**

Ewing sarcoma (ES) is sensitive to systemic therapy, including chemotherapy and anti-angiogenesis Tyrosine Kinase Inhibitors(aaTKIs). However, the prognosis of patients with metastatic disease remains poor. Recurrence or distant metastasis after a complete response (CR) or near-CR due to systemic therapy is not rare.

**Methods:**

We reviewed data from 187 ES patients between 2014–2019 treated at a single institute in China. Patients with extensive lung/pleural metastases (L/Pmeta) who had a CR or near-CR after first- or second-line chemotherapy with or without aaTKIs were retrospectively enrolled. Event-free survival (EFS) and overall survival (OS) were determined using the Kaplan–Meier method. For patients who had L/P recurrence, images were reviewed to define the exact location of each recurrent lesion, compared with the primary L/P lesion before chemotherapy and summarized as the relapse pattern.

**Results:**

Seventeen patients and 21 cases of CR/nCR (5 by VDC/IE, 3 by VIT, and 13 by AVI) were finally analyzed. Median follow-up for surviving patients was 39.6 (range, 14.5–60.9) months. Median EFS and OS were 9.3 (95% confidence interval [CI], 2.0–16.6) months and 37.5 (95% CI, 21.8–53.1) months, respectively. The 2-year EFS was 19% and the 2-year OS was 70.6%, respectively. Most patients (82.4%) received whole lung irradiation (WLI). Lung/pleural relapse occurred in 71.4% (15/21) of CR/nCR cases. Most notably, all recurrent lesions exactly coincided with the original metastatic lesions before chemotherapy (exactly in situ) in 9 of the 15 recurrent cases, which was thus the major relapse pattern, whereas 42.9% had distant metastases other than L/Pmeta.

**Conclusions:**

Survival of ES patients with extensive L/Pmeta remains poor, even if they have a CR after systemic therapy. Recurrence exactly in situ is the major relapse pattern. WLI is not sufficient to prevent local recurrence in lung or pleura. More aggressive local treatment for metastatic lesions is warranted.

## Introduction

Ewing sarcoma (ES) is a small blue round cell tumor derived from primordial mesenchymal stem cells and is generally considered sensitive to chemotherapy. However, the prognosis of ES patients with initial or secondary metastatic disease is relatively poor [1]. Current therapies for patients who present with metastatic disease achieve 6-year event-free survival (EFS) of approximately 28% and overall survival (OS) of approximately 30% [2]. In the clinical trial series Euro-Ewing 99, 2008 [3] and 2012 [4], patients with pulmonary metastases were classified as intermedia risk, designated the Risk 2 pulmonary (R2pulm) group. For this group, 6-year EFS is approximately 40%, better than patients with bone/bone marrow metastases [5]. On the other hand, the prognosis of patients with recurrent or refractory disease is poor, with a 5-year OS after recurrence of 10–15% [1].

For patients with lung/pleural metastases (L/Pmeta), aggressive chemotherapy followed by whole lung irradiation (WLI) is considered to be standard treatment [3, 6, 7]. Most patients with ES are thought to be chemo-sensitive, and 4.2%-33.3% of patients with L/Pmeta had a complete response (CR) to either first- or second-line chemotherapy [5, 8]. Although WLI was given to prevent recurrence in the lung, not all of these complete responders survived longer-term. Secondary tumors refractory to chemotherapy or relapse in the follow-up period occurred in the majority of these former responders and limited their long-term survival.

There have been numerous clinical trials aiming at the prognosis of R2pulm group or recurrent/relapsed (RR) ES patients [1, 5, 8, 9]. However, for those who had extensive L/Pmeta and then completely responded to chemotherapy, there is insufficient high quality data to answer the following questions: 1. How much portion of these patients could be cured at last? 2. If not, what are the relapse patterns in these former responders? To answer these questions, we carried out this retrospective study and reviewed ES patients from one institutes in China.

## Materials and methods

### Patients

We conducted an Institutional Review Board-approved review of all patients with complete medical records diagnosed with ES and treated at Peking University People’s Hospital and Peking University Shougang Hospital between 2014 and 2019. Inclusion criteria were: 1. Histologically-confirmed EWS (but EWSR-FLI 1 translocation by fluorescence in situ hybridization (FISH) was not required); 2.Extensive L/Pmeta with no chance of radical radiotherapy (RT) or resection before multi-drug chemotherapy. Extensive L/Pmeta was defined when it fulfilled at least one of the following criteria: 1) Multiple pulmonary nodules in both lungs, and the largest nodule ≥ 0.5 cm (More than 5 nodules were defined as “multiple” in this study). 2) Pleural effusion and/or multiple pleural nodules; 3. Experiencing a complete response or nearly completely response (CR/nCR)] recorded and then confirmed after 6–8 weeks of multi-drug systemic therapy based on chemotherapy with or without aa-TKIs. CR was defined according to the Response Evaluation Criteria In Solid Tumors 1.1 (RECIST 1.1) guidelines. nCR was defined as each nodule < 3 mm. Exclusion criteria were: 1. Uncontrolled primary lesion during the same chemotherapy regimen: 1) Primary lesion too large for complete resection or radical radiation after induction chemotherapy. 2) Primary lesion progressing during the same regimen. 3) Distant metastasis other than in the lungs or pleura, such as bone, bone marrow, or lymph nodes.

### Treatment

Alternating vincristine/doxorubicin/cyclophosphamide and ifosfamide/etoposide (VDC/IE) q2w were administered as first-line chemotherapy. Irinotecan-based regimens such as vincristine/irinotecan/temozolomide (VIT), or anlotinib, an aa-TKI plus vincristine and irinotecan was used as second-line chemotherapy in recurrent or refractory patients (NCT03359005 and NCT03416517).

For newly-diagnosed patients, surgery was preferred as the local treatment modality unless it was judged to be associated with significant morbidity, in which case RT was recommended. RT doses were 55.8 Gy for gross disease and 50.4 Gy for resected disease with positive margins in three-dimensional conformal therapy (3DCRT). Stereotactic body radiation therapy (SBRT) was also allowed for certain cases.

WLI was given at the end for patients with first-line chemotherapy, or if all pulmonary lesions were < 3 mm in two consecutive CT scans for recurrent or relapsed patients. WLI was given by the 3DCRT technique. The median dose of prescribed radiation was 15 Gy/1.5 Gy/F, once per day; with the dose changing according to the hospital treatment policy from 12 Gy earlier to 15 Gy more recently. Notably, no patients received a boost dose.

### Outcomes

**Fig. 1:**
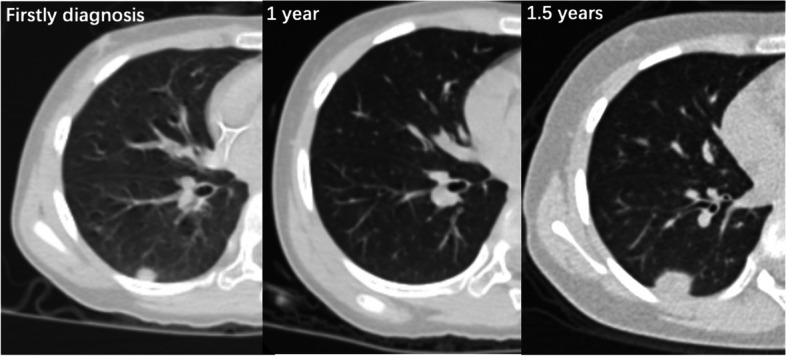
A 9-year-old girl diagnosed with ES of right proximal humerus. She showed multiple pulmonary metastasis at diagnosis at 2016–7-19. At the end of 14 cycles of first-line compressed VDC-IE, CR was confirmed at 2017–6-30. Neither maintenance therapy nor WLI were given. Half a year later, exactly relapse in situ was diagnosed at 2017–12-18

EFS was defined from the date of first confirmation of CR/nCR to the first reported event (recurrence, progression, or death). Patients without an event were censored at the date of last follow-up. OS was defined from the date of first confirmation of CR/nCR to death or the date that they were last known to be alive. Chest computed tomography (CT) images were reviewed by two doctors to define the exact location of each recurrent lesion, compared with the primary L/P lesion before chemotherapy, and the relapse pattern was then summarized according to the following criteria:

- Pattern 1 = All recurrent lesions completely coincide with the original metastatic lesions before chemotherapy (Fig. 1)
- Pattern 2 = Some of the recurrent lesions coincide with the original lesions, but also with some new lesions
- Pattern 3 = All recurrent lesions are completely new lesions

### Statistical analysis

Images were retrospectively reviewed by two independent radiologists. Descriptive statistics including age, sex, time from initial diagnosis to extensive L/Pmeta, regimen of systemic therapy before CR/nCR and lesions with outside L/Pmeta were used to report patient characteristics. EFS and OS were calculated using the Kaplan–Meier method. All statistical analyses were performed in SPSS software package (SPSS Inc, Chicago, IL, USA).

## Results

### Patient characteristics

From July 1st 2014 to July 1st 2019, 17 patients and 21 cases of CR/nCR (5 by VDC/IE, 3 by VIT, and 13 by AVI) were finally analyzed (Table 1). Among these 17 patients, two experienced CR/nCR twice and one patient experienced CR/nCR a third time. Median age at diagnosis of CR/nCR was 16 years (range, 4–39). Of these patients, 70.6% were male, and 23.8% had primary extensive L/Pmeta, while 19.0% suffered L/Pmeta within 12 months of the initial diagnosis of ES, and 57.1% after more than 12 months. The median time from extensive L/Pmeta to first confirmation of CR/nCR was 5.2 months. Median follow-up of surviving patients was 39.6 (range, 14.5–60.9) months.

**Table 1:** Demographics and baseline characteristics (21 CR/nCR in 17 patients^a^)

Patients	Number	%
Age (years; Mean)	16 (range 4 – 39)	-
Sex		
Male	12	70.6%
Female	5	29.4%
EWSR-ETS translocation		
No	0	0
Yes	13	76.5%
Not available	4	23.5%
Primary site of the tumor		
Axial^b^	6	42.9%
Extremities	8	57.1%
Time from initially diagnosis of ES to extensive L/PMeta		
0	5	23.8%
< 12 months	4	19.0%
> 12 months	12	57.1%
Chemotherapy regimen before CR/nCR		
VDC-IE	5	23.8%
VIT	3	14.3%
AVI	13	61.9%
Time from L/PMeta to CR/nCR (months; Mean)	5.2	-
Lesions other than L/PMeta		
none	17	81.0%
Primary bone lesion	4	19.0%

^a^Two patients experienced CR/nCR twice and one experienced CR/nCR a third time

^b^No primary mediastinal EWS was included

### Treatment details and survival

Details of treatments and outcomes for each case are summarized in Table 2, showing that 82.4% patients received WLI. Maintenance therapy was given in 16 cases tailored to the patient at the discretion of the investigator, including chemotherapy, anti-angiogenesis TKI, poly ADP-ribose polymerase (PARP) inhibitor and nonsteroidal anti-inflammatory drugs (NSAIDs).

**Table 2:** Maintenance therapy and survival after CR/nCR

Baseline characteristics				From meta to CR/nCR				Time from CR/nCR to the following conditions (months)			
No	Age	Time to meta	Baseline metastases^a^	Regimen before CR/nCR	Time to CR/nCR	WLI	Maintenance therapy	Status at last follow-up^b^	Last follow up	Local recurrence or metastatis	Death
1	8y	8.9	L	AVI	1.7	Yes	VI	DOD1	-	12.4	30.0
2	21y	10.3	P	AVI	3.6	Yes	CTX + Celecoxib	DOD2	-	6.2	37.5
3	21y	11.7	L	AVI	4.0	Yes	AVI	NED	38.8	-	-
4	23y	15.2	L	AVI	6.3	Yes	VI	AWD	33.4	3.5	-
5	36y	41.7	L	AVI	7.0	Yes	AVI	AWD	32.3	17.2	-
6	12y	8.5	L + P	AVI	5.7	Yes	A	DOD2	-	2.6	22.0
7	18y	21.7	L + P	AVI	5.8	Yes	VI + olaparib	DOD2	-	6.6	14.4
8	8y	18.7	L	AVI	2.8	Yes	A	AWD	33.0	6.5	-
9	39y	29.9	L	AVI	4.2	Yes	AVI	AWD	28.6	15.9	-
10	8y	0.0	L	VDC-IE	3.1	No	CTX + Celecoxib	AWD	63.8	31.5	-
	11y	14.7	L	AVI	7.5	Yes	A	AWD	24.8	22.8	-
11	13y	0.0	L	VDC-IE	6.2	No	No	DOD1	-	7.5	26.4
	15y	23.5	L	VIT	3.5	No	No	DOD1	-	4.1	5.6
12	4y	0.0	L	VDC-IE	4.1	No	No	AWD	57.7	12.9	-
	6y	17.1	L	IE-VI	6.5	No	CTX + Celecoxib	AWD	38.3	17.8	-
	8y	41.4	L	VIT	9.8	No	No	AWD	10.7	8.7	-
13	24y	0.0	L	VDC-IE	5.4	No	No	DOD1	-	9.3	16.8
14	28y	15.3	L	AVI	4.6	Yes	VIT	DOD1	-	2.4	17.2
15	30y	19.7	L	AVI	6.4	Yes	AVI	NED	33.2	-	-
16	8y	18.9	L	AVI	4.1	Yes	VI	DOD2	-	2.0	12.8
17	5y	0.0	P	VDC-IE	6.2	Yes	CTX + Celecoxib	NED	35.4	-	-

Time to meta: From initial diagnosis of EWS to the experience of ExL/PMeta (months)

Time to CR/nCR: From the delivery of first dose of chemotherapy drug to CR/nCR (months)

*WLI* Whole lung irradiation, *AVI* Anlotinib + Vincristine + Irinotecan, *VIT* Vincristine + vincristine + irinotecan, *VDC-IE* Vincristine + doxorubicin + cyclophosphamide / ifosfamide + etoposide, *A* Anlotinib, *VI* Vincristine + irinotecan, *NED* None evidence of disease, *AWD* Alive with disease, *DOD* Dead of disease

^a^L = only lung metastases only; P = only pleural effusion/nodules; L + P = both lung and pleural effusion/nodules

^b^*DOD1* Died of lung/pleural metastases, *DOD2* Died of other reasons regarding to EWS

**Fig. 2:**
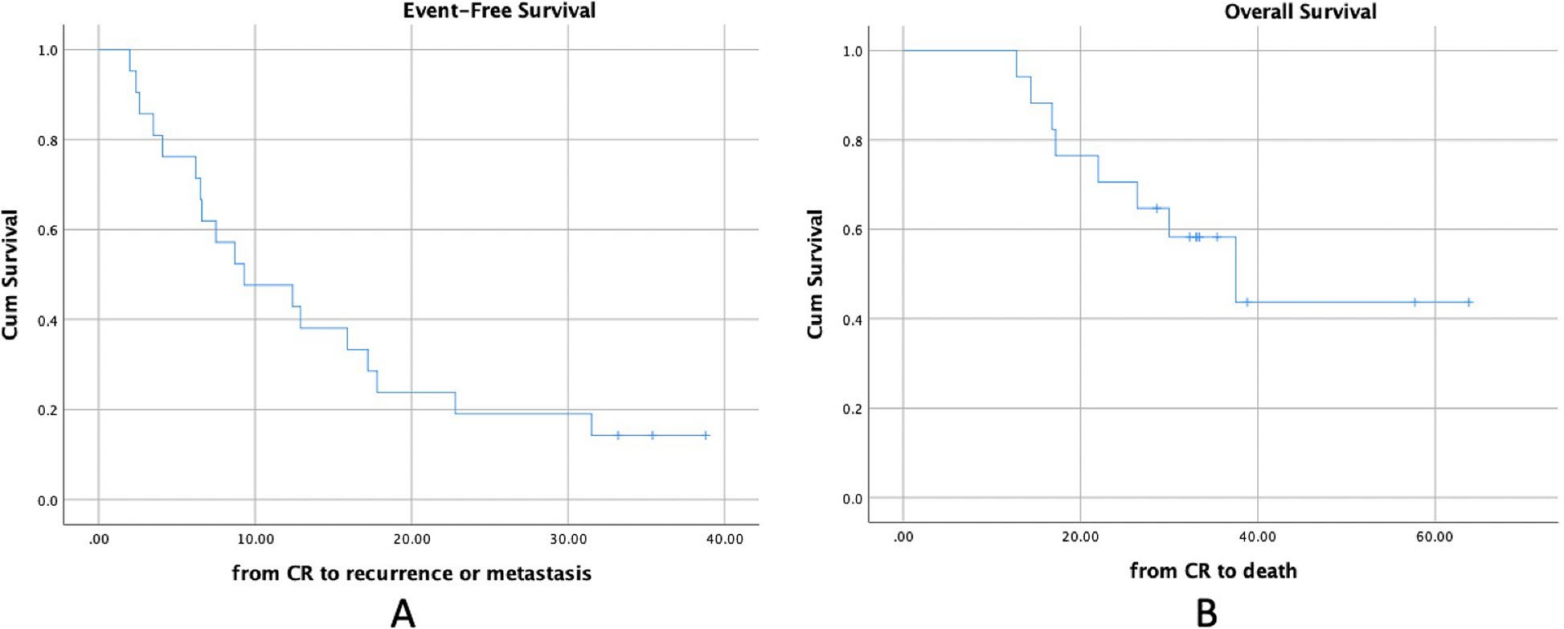
Event free survival and Overall survival calculated from the confirmation of CR/nCR to event or death

**Fig. 3:**
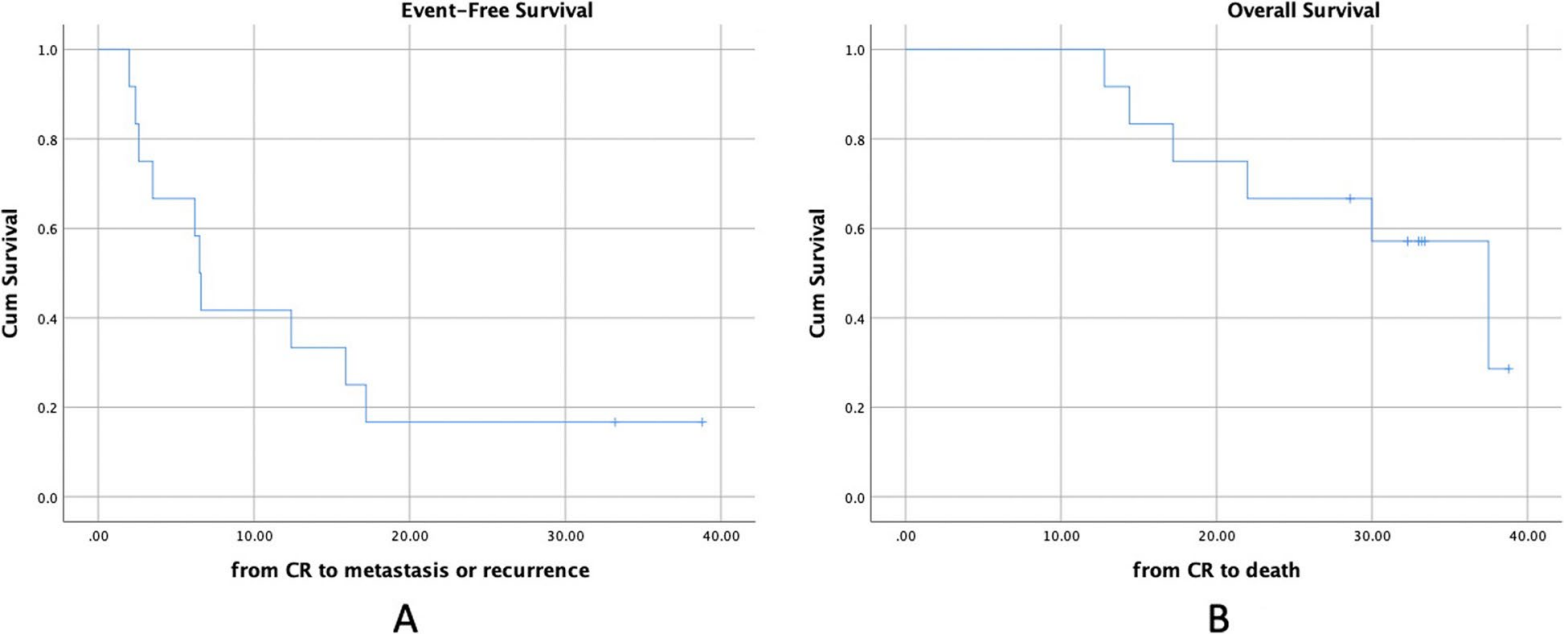
Event free survival and Overall survival calculated from the confirmation of CR/nCR to event or death for 12 relapsed cases

At last assessment, 3 patients had no evidence of disease, 6 were still alive with disease and 8 had already died of disease. For 8 patients died of disease, 4 died of recurrent lung/pleural metastases, while 4 of them died of distant metastases other than lung/pleual lesions. The median EFS and OS were 9.3 (95% CI, 2.0–16.6) months and 37.5 (95% CI, 21.8–53.1) months. The 2-year EFS was 19%. The 2-year OS was 70.6% (Fig. 2). In all 17 patients, 12 were relapsed cases while the other 5 were initially metastatic cases. For the 12 relapsed cases, all of them joined clinical trial NCT03416517 (AVI for relapsed or recurrent EWS). The median EFS and OS were 6.5 (95% CI, 5.8–7.2) months and 37.5 (95% CI, 26.4–48.6) months. The 2-year EFS was 16.7% and OS was 66.7% (Fig. 3).

### Patterns of relapse

Data on the patients who suffered disease relapse are summarized in Table 3. Lung/pleural relapse occurred in 71.4% (15/21) of CR/nCR cases. Most notably, in 9 of the 15 recurrent cases, the locations of all recurrent lesions exactly coincided with the original metastatic lesions before chemotherapy (i.e. exactly in situ). This was the major relapse pattern; however, 4 of these 9 cases also had distant metastases, while the other 5 cases had no lesions outside the lung/pleura. In 2 of the 15 cases with lung/pleural recurrence, some of the recurrent lesions were exactly in situ, but some new lesions in the lungs were also noted. Neither patient had distant metastases concurrently. For the last 4, all recurrent lung/pleural lesions were completely new, and two also had distant metastases as well. For the whole cohort, 42.9% had distant metastases outside of the L/Pmeta.

**Table 3:** Relapse pattern after CR/nCR

No	Whole lung irradiation	Time from CR/nCR to lung/pleural recurrence	Pattern of recurrence	Distant metastasis
1	Yes	12.4	1	No
2	Yes	6.2	1	Yes
4	Yes	3.5	3	No
5	Yes	-		Yes
6	Yes	2.6	1	Yes
7	Yes	6.6	1	Yes
8	Yes	-		Yes
9	Yes	15.9	3	Yes
10	No	31.5	3	Yes
	Yes	22.8	1	No
11	No	7.5	1	No
	No	4.1	2	No
12	No	12.9	1	No
	No	17.8	3	No
	No	No		Yes
13	No	9.3	2	No
14	Yes	2.4	1	No
16	Yes	2.4	1	Yes

Definition of each pattern according to the locations of recurrent lung/pleural lesions

1 = All recurrent lesions completely coincide with the original metastatic lesions before chemotherapy

2 = Some of the recurrent lesions coincide with the original lesions, but also with some new lesions

3 = All recurrent lesions are completely new lesions

## Discussion

In the Children's Oncology Group studies in America, three risk groups are defined at diagnosis based on the location of lesions: patients with localized tumors, patients with lung or pleural metastases only, and patients with extrapulmonary or multiple metastases [4, 10–12]. For ES patients, L/Pmeta were noted in 25% at diagnosis and classified as intermediate risk [13]. The EFS of patients with L/Pmeta has improved in the last three decades, from 36% at 5 years in the German/European Intergroup Cooperative Ewing's Sarcoma Studies (CESS81, CESS86, EICESS92) [5], and 48% at 5 years in the Italian Sarcoma Group and Scandinavian Sarcoma Group ISG/SSG IV Study [14], to 53.6% at 3 years and 47.9% at 8 years in Euro-E.W.I.N.G. 99 or EWING 2008 studies [3]. In the most recent Euro-Ewing 2012 trial, survival data are not available [15] The EFS and OS in our study seemed much worse than in these historical trials. On the one hand, compared to these trials which recruited patients with one or more lesions, the patients in our study had extensive L/Pmeta, equivalent to a higher metastatic load. Patients with unilateral metastases had significantly better outcomes than patients with bilateral metastatic lesions [3, 5]. On the other hand, we enrolled patients failing first-line chemotherapy, which may also partly explain the unsatisfactory results.

The relapse rate was higher in L/Pmeta patients compared with patients with locoregional disease: 50% versus 20–39% [3, 5]. However, previous reports did not mention the location and relapse pattern of L/P recurrence. Our study is the first to investigate the location of recurrent and previous metastatic lesions before systemic treatment (Table 3). As most cases (9/15) in our study relapsed in situ, we conclude that recurrence in situ in L/Pmeta lesions is the major relapse pattern in these patients. A more aggressive local treatment other than WLI is needed. WLI may be used prophylactically in localized ES, rather than therapeutically in L/Pmeta, even for patients with a CR/nCR [16–18].

It is commonly accepted that ES prognosis is critically determined by the degree of adequacy of local control of the primary tumor and the efficacy of systemic chemotherapy to eradicate metastatic disease [12]. According to previous reports, lesions in L/Pmeta showed adequate responses during systemic treatment and CR/nCR was not rare. Whole lung irradiation improved outcome in ES patients with lung lesions by 5–20%, even when complete resolution of overt pulmonary metastatic disease had been achieved with chemotherapy [3, 6, 16, 19, 20]. Compared to the high dose of 55-60 Gy in radical radiation for ES lesions, the dose used for WLI is rather low considering the tolerance of normal pulmonary tissue. The higher rate of L/P recurrence might be partially explained by repopulation of the pulmonary space from subclinical metastases that were not eradicated completely by systemic treatment and low-dose WLI [20].

For most sarcoma patients, surgical resection of primary or metastatic lesions is important for long-term survival, as in osteosarcoma or chondrosarcoma. However, in ES, it was noted more than 20 years ago that pulmonary resection of metastatic lesions failed to improve survival [21]. This is in accordance with the high local recurrence rate for L/Pmeta even after adequate chemotherapy. Notably, the failure of surgical resection in L/P lesions in ES may be explained by inadequate resection, rather than surgery itself, given the fact that most L/P lesions were too small to resect after successful systemic treatment. Although ES is considered to be chemo-sensitive compared with other sarcomas, the biological behavior of ES still follows the classical pathway of resistance development in solid tumors and requires aggressive local treatment, but does not conform to the lymphoid and haematopoietic tissue scenario, such as in lymphoma or leukemia which totally relies on systemic treatment. These data support the proposal that more aggressive local treatment strategies, not only effective systemic treatments, should be applied to all involved lesions such as SBRT.

Our study also have some limitations. First, as a single-center retrospective study, we only enrolled 17 patients and 21 cases in the paper. The result may be not exact due to the small number of sample. Although we demonstrate that recurrence exactly in situ is the major relapse pattern, the portion of each pattern according the defination in our study may differs if more samples are analyzed. Second, aaTKIs have been used as part of chemotherapy in second line treatment. The mechanism of most aaTKIs was to cut off the blood supply to tumor resulting in hypoxia–ischemia necrosis. Although CR/nCR was noticed per imaging, a few active tumor cells may remain subclinically in the necrotic tissues. We listed the regimen of systemic therapy in Table 2, but did’t have engouh samples for further COX analysis to define if the use of aaTKIs had an impact on recurrence. Third, although EWSR rearrangedment was confirmed in all 13 patients, the other 4 patients did not have enough specimen for further molecular test. Given the retrospective nature of this study, we failed to get more specimen from biopsy or surgical resection. We indeed had a risk to include so-called Ewing-like sarcoma in our analysis.

## Conclusions

Survival of ES patients with extensive L/Pmeta remains poor, even after they have a complete response to systemic therapy. Recurrence exactly in situ is the major relapse pattern. WLI is not sufficient to prevent local recurrence in lung or pleura. More aggressive local treatment for metastatic lesions is warranted.

## Data Availability

The datasets used and/or analyzed during the current study are available from the corresponding author on reasonable request.

## References

[1] Stahl M (2011). Risk of recurrence and survival after relapse in patients with Ewing sarcoma. Pediatr Blood Cancer.

[2] Miser JS (2007). Treatment of metastatic Ewing sarcoma/primitive neuroectodermal tumor of bone: evaluation of increasing the dose intensity of chemotherapy–a report from the children's oncology group. Pediatr Blood Cancer.

[3] Dirksen U (2019). High-dose chemotherapy compared with standard chemotherapy and lung radiation in Ewing sarcoma with pulmonary metastases: results of the european ewing tumour working initiative of national groups, 99 trial and EWING 2008. J Clin Oncol.

[4] Anderton J (2020). International randomised controlled trial for the treatment of newly diagnosed EWING sarcoma family of tumours - EURO EWING 2012 Protocol. Trials.

[5] Paulussen M (1998). Ewing's tumors with primary lung metastases: survival analysis of 114 (European Intergroup) Cooperative Ewing's Sarcoma Studies patients. J Clin Oncol.

[6] Elghazawy H (2020). Whole lung irradiation for completely responding pulmonary metastases in pediatric Ewing sarcoma. Future Oncol.

[7] Casey DL (2014). Whole lung irradiation for adults with pulmonary metastases from Ewing sarcoma. Int J Radiat Oncol Biol Phys.

[8] Xu J (2021). Anlotinib, vincristine, and irinotecan for advanced ewing sarcoma after failure of standard multimodal therapy: a two-cohort. Phase Ib/II Trial Oncologist.

[9] Xu J (2019). Management of recurrent or refractory Ewing sarcoma: a systematic review of phase II clinical trials in the last 15 years. Oncol Lett.

[10] Felgenhauer JL (2013). A pilot study of low-dose anti-angiogenic chemotherapy in combination with standard multiagent chemotherapy for patients with newly diagnosed metastatic Ewing sarcoma family of tumors: a Children's Oncology Group (COG) phase II study NCT00061893. Pediatr Blood Cancer.

[11] Pappo AS, Dirksen U (2018). Rhabdomyosarcoma, Ewing Sarcoma, and other round cell sarcomas. J Clin Oncol.

[12] Gaspar N (2015). Ewing sarcoma: current management and future approaches through collaboration. J Clin Oncol.

[13] Balamuth NJ, Womer RB (2010). Ewing's sarcoma. Lancet Oncol.

[14] Luksch R (2012). Primary metastatic Ewing's family tumors: results of the Italian Sarcoma Group and Scandinavian Sarcoma Group ISG/SSG IV Study including myeloablative chemotherapy and total-lung irradiation. Ann Oncol.

[15] Brennan B, Kirton L, Marec-Berard P, et al. Comparison of two chemotherapy regimens in Ewing sarcoma (ES): Overall and subgroup results of the Euro Ewing 2012 randomized trial (EE2012). J Clin Oncol. 2020;38(15_suppl):11500–11500.

[16] Spunt SL (2001). Selective use of whole-lung irradiation for patients with Ewing sarcoma family tumors and pulmonary metastases at the time of diagnosis. J Pediatr Hematol Oncol.

[17] Marinova L (2015). Protective, elective lung irradiation in non-metastatic Ewing's sarcoma. Radiat Prot Dosimetry.

[18] Nesbit ME (1990). Multimodal therapy for the management of primary, nonmetastatic Ewing's sarcoma of bone: a long-term follow-up of the First Intergroup study. J Clin Oncol.

[19] Paulussen M, Primary metastatic (stage IV) Ewing tumor: survival analysis of 171 patients from the EICESS studies (1998). European intergroup cooperative ewing sarcoma studies. Ann Oncol.

[20] Ronchi L (2018). Whole lung irradiation in patients with osteosarcoma and ewing sarcoma. Anticancer Res.

[21] Heij HA (1994). Prognostic factors in surgery for pulmonary metastases in children. Surgery.

